# MeDIP-seq and nCpG analyses illuminate sexually dimorphic methylation of gonadal development genes with high historic methylation in turtle hatchlings with temperature-dependent sex determination

**DOI:** 10.1186/s13072-017-0136-2

**Published:** 2017-05-19

**Authors:** Srihari Radhakrishnan, Robert Literman, Beatriz Mizoguchi, Nicole Valenzuela

**Affiliations:** 1 Bioinformatics and Computational Biology Program, Iowa State University, Ames, IA 50011 USA; 2 Ecology and Evolutionary Biology Program, Iowa State University, Ames, IA 50011 USA; 3 Interdepartmental Genetics and Genomics Program, Iowa State University, Ames, IA 50011 USA; 4 Department of Ecology, Evolution and Organismal Biology, Iowa State University, 251 Bessey Hall, Ames, IA 50011 USA

**Keywords:** Sex-specific thermosensitive DNA methylation, Genome-wide normalized CpG content, MeDIP sequencing, Temperature-dependent and genotypic sex determination, Turtle gonadal embryonic development, Ecological genomics, Epigenetic modification, Phenotypic plasticity, Sexual development, Reptile vertebrate

## Abstract

**Background:**

DNA methylation alters gene expression but not DNA sequence and mediates some cases of phenotypic plasticity. Temperature-dependent sex determination (TSD) epitomizes phenotypic plasticity where environmental temperature drives embryonic sexual fate, as occurs commonly in turtles. Importantly, the temperature-specific transcription of two genes underlying gonadal differentiation is known to be induced by differential methylation in TSD fish, turtle and alligator. Yet, how extensive is the link between DNA methylation and TSD remains unclear. Here we test for broad differences in genome-wide DNA methylation between male and female hatchling gonads of the TSD painted turtle *Chrysemys picta* using methyl DNA immunoprecipitation sequencing, to identify differentially methylated candidates for future study. We also examine the genome-wide nCpG distribution (which affects DNA methylation) in painted turtles and test for historic methylation in genes regulating vertebrate gonadogenesis.

**Results:**

Turtle global methylation was consistent with other vertebrates (57% of the genome, 78% of all CpG dinucleotides). Numerous genes predicted to regulate turtle gonadogenesis exhibited sex-specific methylation and were proximal to methylated repeats. nCpG distribution predicted actual turtle DNA methylation and was bimodal in gene promoters (as other vertebrates) and introns (unlike other vertebrates). Differentially methylated genes, including regulators of sexual development, had lower nCpG content indicative of higher historic methylation.

**Conclusions:**

Ours is the first evidence suggesting that sexually dimorphic DNA methylation is pervasive in turtle gonads (perhaps mediated by repeat methylation) and that it targets numerous regulators of gonadal development, consistent with the hypothesis that it may regulate thermosensitive transcription in TSD vertebrates. However, further research during embryogenesis will help test this hypothesis and the alternative that instead, most differential methylation observed in hatchlings is the by-product of sexual differentiation and not its cause.

**Electronic supplementary material:**

The online version of this article (doi:10.1186/s13072-017-0136-2) contains supplementary material, which is available to authorized users.

## Background

Epigenetic modifications are heritable changes to the DNA that do not change the nucleotide sequence. Among them, DNA methylation is a biochemical process that adds methyl groups to cytosine or adenine nucleotides. Methylated DNA alters gene expression by preventing transcription factor binding [1] or by sometimes favoring the binding of repressors [2, 3]. The regulatory role of DNA methylation is widespread across eukaryotes where it mediates development, environmental responses and disease [4–7]. In animals, the addition of methyl groups occurs on CpG dinucleotides (cytosine linked to a guanine by a phosphate group) within genes in invertebrates [8] and across genic and intergenic regions in vertebrates [9]. Importantly, changes in DNA methylation levels have been linked to the regulation of phenotypic plasticity [10–12]. Temperature-dependent sex determination (TSD) represents a textbook example of phenotypic plasticity (a thermal polyphenism), where individuals with identical genotypes can develop alternative phenotypes (male or female) based on environmental cues [13, 14]. Differential methylation of two genes in the sex-determining pathway has been experimentally identified in a few TSD vertebrates (a fish, a turtle and alligator) [15–17] and in other genes during temperature-induced sex reversal in a fish with a mixed sex-determining system (ZZ/ZW GSD and TSD) [18]. However, the extent to which TSD plasticity is mediated by DNA methylation in turtles remains unclear. As an initial step to address this question, it is necessary to characterize the level of methylation in the genome of TSD turtles and to test whether methylation patterns differ in males and females as would be expected if temperature induces sex-specific methylation profiles [18]. Additionally, if TSD is mediated by DNA methylation of the regulatory network of gonadal development, it would be expected that genes in this network would be the target of differential methylation. And if differential methylation of this network has occurred over evolutionary time, these elements should display a signature left by historic methylation at the DNA sequence level [19, 20].

In silico techniques have been used to estimate historic DNA methylation patterns in animals by measuring the normalized CpG content (or nCpG), i.e., the ratio of the CpG dinucleotide abundance observed at particular genomic regions compared to that expected at random based on the frequency of cytosines and guanines present in the genome [CpG(O/E) = CpG observed/expected] [21]. This value of nCpG is used as a proxy for DNA methylation since (a) DNA methylation is almost entirely targeted to CpG dinucleotides in animals [22], and (b) 5-methylcytosine has the tendency to undergo spontaneous deamination which converts it to thymine leaving a footprint that reflects historic methylation levels [19, 20, 23, 24]. Therefore, nCpG is inversely correlated with the extent of DNA methylation such that in hypermethylated regions (where cytosines within methylated CpGs have been converted to thymine) the nCpG is less than one. On the other hand, an nCpG ratio equal to 1 is indicative of no deviation from random expectation, while a value greater than one indicates hypomethylated regions.

The genome-wide patterns of animal DNA methylation vary within and between invertebrates and vertebrates. For instance, in multiple ant species the genomic distribution of nCpG values is unimodal and centered around 1 [25], whereas the nCpG distribution in honeybee and pea aphid genic regions is bimodal with one peak centered at 0.5 and the other at 1 [26]. Among vertebrates, the promoter regions of eutherian mammals, opossum, chicken, lizard, frog and fish also show a bimodal pattern, such that genes with lower nCpG content (LCG promoters) undergo higher methylation and are linked to tissue-specific expression, and those with higher nCpG content (HCG promoters) are hypomethylated and are linked to broad patterns of gene expression [27, 28]. In contrast, the nCpG content of promoters in platypus (and the urochordate sea squirt) is unimodal [27, 29].

Other studies across various vertebrates reported a genome-wide average CpG ratio of ~25% for mammals and birds and of ~35% for fish and amphibians using alternative approaches such as restriction enzyme assays and HPLC [19, 24, 30]. Because of earlier difficulties to study nCpG distribution in reptilian DNA sequences [31], data at this level are scarce for reptiles. Indeed, pioneering work on reptilian methylation levels focused more heavily on global levels [24, 31], and to our knowledge, only one study examined nCpG content and DNA methylation [27] and another study examined non-methylated islands [32] in anole lizards. Thus, the pattern of genome-wide nCpG distribution in turtles and in TSD vertebrates remains unknown at the DNA sequence level. This is critical given that nCpG not only reflects historic DNA methylation, but is a factor that mediates current DNA methylation levels in ways that influence transcription levels.

Here, we test for broad differences in genome-wide DNA methylation between male and female hatchling gonads of the TSD painted turtle, *Chrysemys picta*, to identify methylated regions of interest that may be important for phenotypically plastic sexual development, which would represent candidates for further analyses in future studies. A similar approach was used to investigate the association of DNA methylation and TSD in a ZZ/ZW + TSD fish by examining DNA methylation in mature individuals [18]. We concentrate our search on genes known to regulate vertebrate primary sexual development [33], as well as genes that by their nature may help mediate TSD sex-specific development, such as genes of the epigenetic machinery [34], hormonal pathways [35] or general sensing responses [36]. Second, we examine the genome-wide nCpG distribution in the painted turtle genome [37, 38] and compare it to that of other vertebrates. We then test whether this index predicts the DNA methylation landscape in turtle gonads and also test for a signature of historic methylation on gene regulators of vertebrate sexual development. Thus, our study provides the first insight into the association between nCpG content and differential methylation in any TSD vertebrate.

## Results

### Genome-wide CpG distribution is bimodal in turtle promoters, introns, exons and intergenic regions

**Fig. 1:**
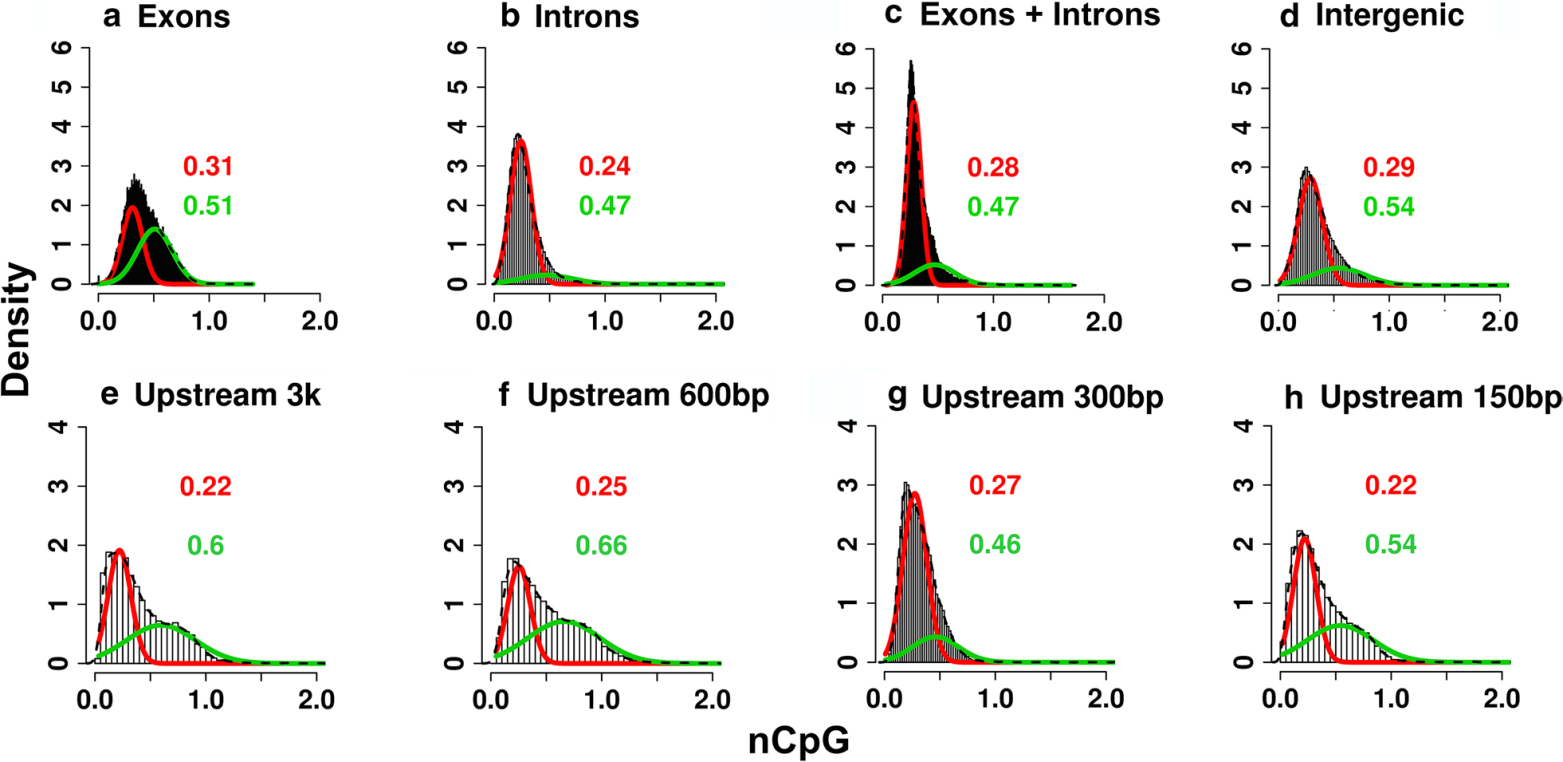
Distribution of normalized CpG (nCpG) content in the *Chrysemys picta* genome by region. **a** Exons only (CDS), **b** introns only, **c** exons and introns, **d** intergenic regions and **e**–**h** at 3000, 600, 300 and 150 bases upstream of exon 1. Fitted Gaussian density curves for the bimodal distribution along with their respective peak values are indicated in *red* and *green*

In all genomic regions, the nCpG was much lower than the expected ratio of 1, predicting that a significant fraction of the painted turtle genome is methylated. Notably, for the exons the overall distribution of nCpG was centered at 0.35, whereas the distributions for the rest of the profiled regions were centered at 0.25. Importantly, although at first glance the profiles of genome-wide normalized CpG content (nCpG) appeared unimodal, statistical analyses fitting mixture models to the data [39] revealed higher support for bimodal distributions across gene bodies (exons plus introns), exons and introns individually, and the intergenic regions (*p* value of likelihood ratio test <0.00001) (Fig. 1a–d). Interestingly, bimodality was even more obvious in the nCpG profiles of promoter regions measured as 3000, 600, 300 or 150 bases upstream of exon 1 (*p* value of likelihood ratio test <0.00001) (Fig. 1e–h).

### A substantial portion of the turtle genome is methylated

We used MeDIP-seq to characterize the DNA methylation landscape broadly by profiling differentially methylated regions rather than individual cytosines [40] in two pools of gonads from five male hatchlings each and two pools from five female hatchlings each (Table 1). Over 98% of the MeDIP-seq reads from the male and female hatchling gonads mapped to the *C. picta* genome [37]. The methylome analysis uncovered ~2.95 million methylated 500-bp windows, totaling 1.48 gigabases in size, or ~57% of the assembled genome [37], and overlapping with 17,646 genes. This corresponds to 78% of the CpG nucleotides in the genome. A total of 40% of the methylated windows fall within gene bodies which is significantly less than the 46% located within 50-kb-upstream sequences that have potential regulatory functions (permutation test *p* value =0.001). The remaining 14% of methylated windows fall in intergenic regions outside of gene bodies or outside sequences 50 kb upstream of all genes.

**Table 1:** Illumina library statistics for MeDIP sequencing of *Chrysemys picta* hatchling gonads

Sex (incubation temp)	Library size	% Mapped reads
Male (26 °C) Lib1	137,159,464	98.85
Male (26 °C) Lib2	138,462,674	98.80
Female (31 °C) Lib1	126,682,102	98.83
Female (31 °C) Lib2	163,323,313	98.87

### Sexually dimorphic DNA methylation varies by gene region

**Fig. 2:**
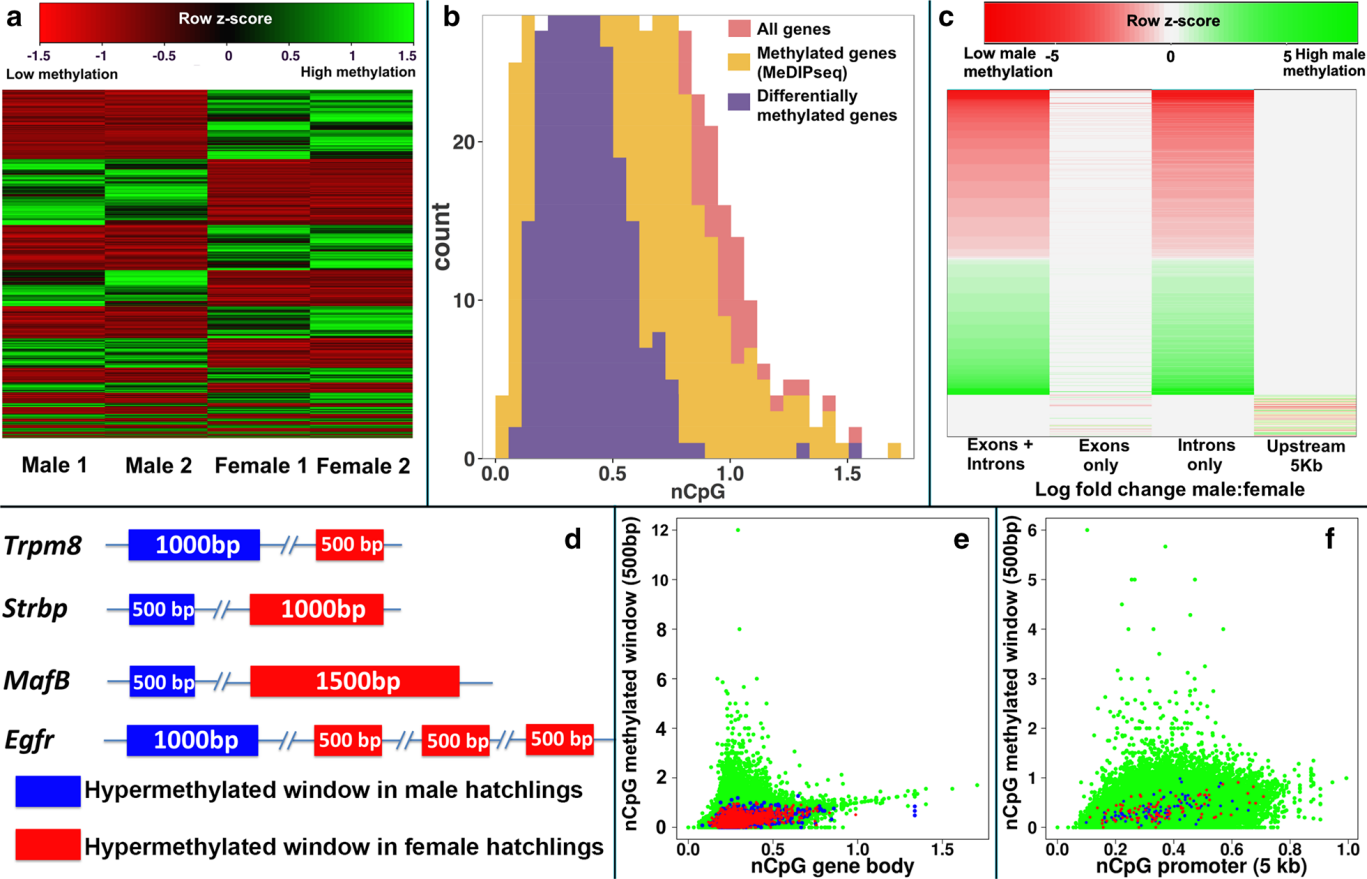
**a** RPKM heatmap of differentially methylated genes in *Chrysemys picta* (*rows*) clustered by mean methylation level per gene. Methylation levels were scaled to [−1.5, 1.5] to indicate genes undergoing high (*green*) and low (*red*) relative methylation. **b** Normalized CpG content of all annotated genes (*red*), experimentally verified to be methylated using MeDIP-seq (*yellow*) and differentially methylated (*purple*). **c** Fold change in methylation (*red*: hypermethylated in female; *green* hypermethylated in male) as seen in gene bodies (exons + introns), exons only, introns only and promoters. **d** Examples of genes possessing multiple windows that displayed sex-specific methylation. **e**, **f** Scatterplot of normalized CpG content (nCpG) in methylated windows occurring (**e**) in gene bodies relative to nCpG of gene bodies and **f** in promoters relative to nCpG of the complete promoter sequence (~5 kb upstream). Differentially methylated windows in hatchlings are overlaid, with those hypermethylated in males indicated in *blue*, and those hypermethylated in females indicated in *red*

**Fig. 3:**
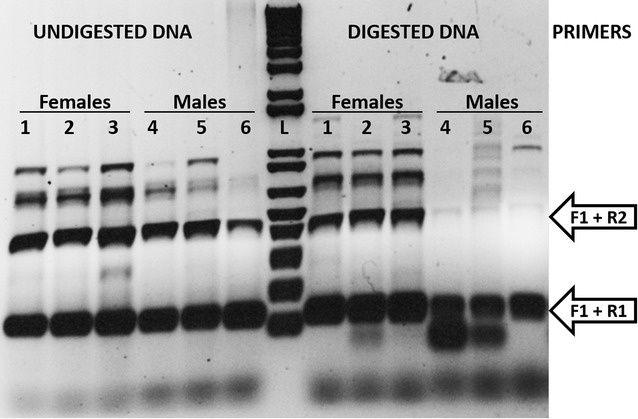
Validation of sexually dimorphic DNA methylation of the *fezf2* gene in *Chrysemys picta* hatchling gonads by methylation-sensitive restriction enzyme PCR. DNA from three females (1, 2 and 3) and three males (4, 5 and 6) was digested or not with HpaII (HpaII cuts unmethylated DNA). Expected size of the PCR amplicons from PCR primers F1 plus R1, or F1 and R2 are indicated by the *arrows* (non-specific PCR products were also obtained). L = DNA ladder (1 kb plus). Amplification of the expected fragment in females and not males using the F1 + R2 primers confirms the hypermethylation of *fezf2* in females detected using the MeDIP-seq data

MeDIP-seq results from the biological replicates revealed strong differences between males and females above and beyond the differences between individual replicates (Fig. 2). Positive (methylated DNA) and negative (unmethylated DNA) controls were used during the MeDIP step and ensure that the MeDIP worked properly, such that the observed variation between the two replicates of the same sex likely reflects natural population-level variation in methylation among individuals. This was observed also using PCR of DNA of multiple males and females digested or not with a restriction enzyme sensitive to DNA methylation (Fig. 3). Our MeDIP-seq analysis revealed 5647 differentially methylated windows between the sexes. Of these, 3076 windows were hypermethylated in females (in 2414 genes) and 2571 windows in males (in 2086 genes) (Fig. 2a). The log-fold change in methylation between the sexes was highest in introns, followed by promoter sequences and finally exons (Fig. 2c). Additionally, differential methylation of exons was around half that of promoters (there were 536 differentially methylated windows in promoter sequences vs. 281 in exons). Finally, 541 differentially methylated genes contained multiple windows with contrasting sex-specific methylation, such that some windows were hypermethylated in male hatchlings and other windows within the same gene were hypermethylated in females (Fig. 2d; Additional file 1: Table S1).

### Potential mediators of thermal transduction and regulators of sexual development are differentially methylated

While no particular GO terms were significantly enriched in the MeDIP-seq data after controlling for false discovery (Additional file 1: Tables S2, Additional file 1: Table S3), we detected some interesting pathways and genes with differential methylation. We focused our attention on reptilian homologs of genes that govern mammalian gonadogenesis [33, 41] and on genes involved in painted turtle gonadal development [42] whose biological functions may help convert the temperature signal into the sexual fate of TSD embryos (Additional file 1: Table S4, Additional file 1: Table S5, Additional file 1: Table S6, Additional file 1: Table S7, Additional file 1: Table S8, Additional file 1: Table S9, Additional file 1: Table S10, Additional file 1: Table S11). Full list of differentially methylated genes and GO pathways are presented in Additional file 1: Table S12, Additional file 1: Table S13, Additional file 1: Table S14). Among these were a number of kinases, androgen-/estrogen-related genes, histone- and ubiquitin-related genes, heat shock and transient potential receptor genes that displayed sexually dimorphic methylation. Interestingly, members of the *Wnt* signaling pathway and genes involved in transcriptional regulation tended to be hypermethylated in males relative to females, whereas genes involved in cell and neuron differentiation tended to be hypermethylated in female hatchlings. Importantly, 13 out of 53 reptilian homologs of genes involved in mammalian gonadogenesis [33, 41] were differentially methylated. For instance, genes that regulate testicular formation, some of which are highly expressed during embryogenesis at male-producing temperature in TSD turtles, including *Amh*, *Ar*, *Gata4*, *Lhx1*, *Lhx9* and *Sf1* [42–46], were significantly hypermethylated in female hatchlings. In contrast, genes important in ovarian formation in mammals, such as *Wnt4* and *Emx2* [47, 48], were hypermethylated in males. Differential methylation also varied by genic region, perhaps reflecting differences in the type of regulation that DNA methylation might exert in several genes in the sexual development network. For instance, while some of these genes exhibited hypermethylation in the promoter regions near the 5′ end, others were hypermethylated in their gene bodies, mostly in their intronic sequences. Among them, *Wt1* exhibited three hypermethylated intronic windows in male hatchlings. Yet other genes, such as *Lhx1* and *Gata4,* show female hypermethylation in both promoter (1 window each) and intronic sequences (two and three windows, respectively).

### CpG content is a good in silico predictor of DNA methylation

There is no significant difference between the number of genes predicted to be methylated using the nCpG index and the number of genes identified as methylated by MeDIP-seq (two-sample Kolmogorov–Smirnov test, *p* = 0.1931), suggesting that in silico predictions from the nCpG index are fairly accurate. Specifically, 94% of all methylated gene bodies detected experimentally via MeDIP-seq had an in silico nCpG value of 0.5 or lower, thereby showing a strong association between CpG depletion and actual methylation. Further, 98.7% (16,884 genes) of the 17,104 genes with nCpG ≤ 0.5 are methylated. Only 75 out of 17,646 methylated genes show an nCpG ≥ 0.8, including 36 out of 90 methylated tRNA genes.

### Differentially methylated genes suffered greater historic methylation

Interestingly, the nCpG content of the differentially methylated genes was significantly lower (mean = 0.282, range = 0–1.27) than for all methylated genes (mean = 0.306, range = 0–12) identified by MeDIP-seq (resampling test *p* value =0.001) (Fig. 2b), indicating that genes displaying sexually dimorphic methylation show a signature of higher historic DNA methylation. This pattern was observed in gene bodies (comprising of exons and introns) as well as promoters (Fig. 2e, f), suggesting that differential methylation of hatchling gonads was restricted to the genomic regions of greater CpG depletion.

### DNA methylation appears associated with expression of some genes but not all

In the absence of hatchling transcriptomes, we leveraged gonadal transcriptomes of painted turtle embryos incubated at male-producing temperature (MPT) and female-producing temperature (FPT) that we obtained in another study [42], and uncovered potential candidates for future study when we compared the methylome signatures in hatchlings and differential transcription patterns in late-stage embryos (stage 22). Namely, some genes overexpressed in male embryos were hypermethylated in female hatchlings (58 out of 394), and some genes overexpressed in female embryos were hypermethylated in male hatchlings (40 out of 754) (Additional file 1: Table S15). Thus, indirect evidence was detected suggesting that DNA methylation of numerous genes might mediate their sexually dimorphic transcription and that this influence may not be global but a gene-by-gene effect where some genes may be affected while others are not. A direct test also revealed the lack of such global effect, as the Fisher exact test on the contingency table of genes methylated or not that were differentially expressed or not was not significant (*p* > 0.9999). However, we note that while intriguing, the finding of gene-by-gene effects should be taken with caution given the difference in life stage between the transcriptomic and methylomic data.

### Repeat elements abound nearby methylated genes and share identical pattern of differential methylation

**Fig. 4:**
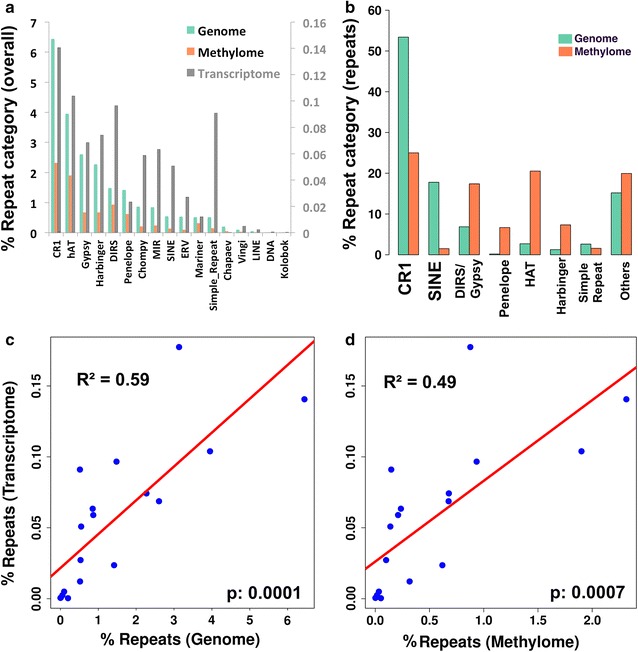
*Chrysemys picta* repeat abundance. **a** Relative abundance of repeat categories in the *Chrysemys picta* genome [37], and their relative abundance in the hatchling gonadal methylome (this study) and embryonic gonadal transcriptome [42] as a fraction of the genome. Note that repeat abundance in the transcriptome is plotted in *gray* and scaled by the right-hand axis for visualization purposes. **b** Relative abundance of various repeat categories within the fraction of repeats present in the *C. picta* genome [37] versus the methylome (this study). **c** Regression of transcriptomic repeat abundance as a function of repeat abundance in the genome (*p* = 0.0001) and **d** in the methylome (*p* = 0.0007). Abundance of repeats in the transcriptome is slightly better explained by their genomic abundance (*R* ^2^ = 0.59) than by their abundance in the methylome (*R* ^2^ = 0.49)

Because repetitive DNA sequences such as transposable elements can be subject to silencing by DNA methylation [49] which could affect nearby genes, we analyzed the repeat content of the methylome. RepeatMasker analyses revealed that around 40% of the methylome consists of repeats (which represent 9.25% of the CPI genome—Additional file 1: Table S16), with significant representation from the CR1 and HAT repeat categories (~45% of the methylome repeats). CR1 repeats were also the most abundant in the *C. picta* gonadal transcriptome [42] (Fig. 4a; Additional file 1: Table S16). Furthermore, the relative abundance among repeat categories as a fraction of the genome (Additional file 1: Table S16) did not differ significantly between the genome, methylome and transcriptome (pairwise Kruskal–Wallis tests; all *p* values >0.453, Fig. 4a), even though the absolute abundance of each category and of all repeats combined varied (e.g., 25, 9 and 1% of the genome, methylome and transcriptome consisted of repeats, respectively—Additional file 1: Table S16). Additionally, methylated repeats were significantly more concentrated around 95% of all methylated genes (resampling test *p* value =0.001) and relatively scarce around non-methylated genes (Table 2). Of these, HAT repeats were the most abundant. Methylated repeats were also common (although significantly less so) in the vicinity of differentially methylated genes (~80% instead of 95%; permutation test *p* value =0.001). Of these, DIRS repeats were the most common. Interestingly, the direction of the sex-specific methylation was identical for 70% of the differentially methylated genes and their neighboring repeats (Table 2).

**Table 2:** Overabundance of methylated repeats upstream of the transcription starting site of differentially and non-differentially methylated genes compared to non-methylated genes in *Chrysemys picta* hatchlings

Number (#) and percentage (%) of methylated repeats	Number of gene bodies with methylated repeats at three distances of start codon		
	1 kb	5 kb	10 kb
Among all 17,646 methylated genes	16,791 (95.1%)	17,030 (96.5%)	17,202 (97.5%)
Among all 433 non-methylated genes	29 (6.7%)	90 (20.8%)	161 (37%)
Among the 2086 male-hypermethylated genes	1650 (79%)	1656 (79.3%)	1662 (79.7%)
Among the 2414 female-hypermethylated genes	1949 (80.7%)	1959 (81.1%)	1961 (81.2%)
Among the 840 methylated genes of interest (Additional file 1: Table S1, Additional file 1: Table S2, Additional file 1: Table S3, Additional file 1: Table S4, Additional file 1: Table S5, Additional file 1: Table S6, Additional file 1: Table S7, Additional file 1: Table S8)	822 (97.8%)	828 (98.6%)	831 (98.9%)
Male-hypermethylated repeat windows located near male-hypermethylated genes	681/946 (72%)	706/973 (72.6%)	718/989 (72.6%)
Female-hypermethylated repeat windows located near female-hypermethylated genes	801/1032 (77.6%)	816/1050 (77.7%)	825/1064 (77.5%)

Repeats were overrepresented upstream of all methylated regions examined (Chi-square test *P* < 0.00001 in all cases) but not upstream of non-methylated genes

Importantly, significantly more genes that were differentially methylated in hatchlings and differentially transcribed in stage 22 embryos were nearby methylated repeats than genes equally expressed in male and females (permutation test *p* value =0.001). The distribution of categories of methylated repeats did not differ between the differentially and non-differentially expressed genes. Using regression, we evaluated the effect of DNA methylation on repeat silencing, by assessing the repeat transcription level as a function of repeat abundance in the genome and as a function of repeat abundance in the methylome. In both cases, the relationship was highly significant and explained a significant proportion of variation in repeat transcription level (repeat abundance in genome: slope = 0.0239, *p* = 0.0001, *r* ^2^ = 0.59; repeat abundance in methylome: slope = 0.0569, *p* = 0.0007, *r* ^2^ = 0.49), although the variation in repeat expression itself was small (Fig. 4c, d). Further, multiple regression analyses with all variables combined did not improve the explanatory power significantly, implying that repeat transcription, repeat methylation status and repeat genomic abundance are tightly linked.

## Discussion

Genomic approaches are advancing our understanding of phenotypic plasticity at unprecedented rates, including the role that DNA methylation plays in mediating plastic responses to environmental inputs [10, 11, 21, 50]. Here we tested whether regulators of vertebrate sexual development (and of other sensing and epigenetic responses) are subjected to differential methylation in male and female hatchlings of a turtle with phenotypically plastic sex determination (*C. picta*) lacking sex chromosomes [51]. Based on these data, we identified candidate genes that may mediate TSD epigenetically. We also characterized the genome-wide nCpG distribution in the painted turtle genome, the first such analysis in reptiles and TSD vertebrates, and found that this proxy predicts reasonably well the methylation levels estimated using MeDIP-seq. Further, nCpG profiles helped assess historic methylation levels of genes regulating vertebrate sexual development. Below we highlight our most important observations and propose working hypotheses to guide further research. Our sex-specific methylomes represent an important genomic resource to aid investigations of the epigenetic regulation of environmental responses.

Although bisulfite sequencing is the gold standard for methylation studies requiring single-nucleotide resolution, MeDIP-seq is appropriate for studies seeking to profile broader patterns of DNA methylation rather than individual cytosines [40], as was our goal. MeDIP-seq provides methylation profiles at a resolution of 150–200 bp, signals tend to concentrate on CpG-rich genomic regions with high methylation levels, and the methylome information can be comprehensive [52], less sequence-biased and fairly concordant with bisulfite-sequencing data for genomes similar in size to those of turtles [37, 52, 53]. The reduction in genomic complexity afforded by MeDIP-seq is also advantageous for taxa with large genomes as is the case of the painted turtle [37, 38]. It should be noted that methylation levels are correlated with 1 kb of neighboring CpG sites [54]. Nonetheless, future bisulfite sequencing will permit evaluating methylation patterns at higher resolution, particularly in non-CpG genomic regions, which was precluded in our study. But importantly, given that methylation status may be more stable at the level of DNA domains rather than at single nucleotides [55], MeDIP-seq snapshots do contain highly valuable information as discussed below.

## Bimodal distribution of nCpG turtle genomes matches most vertebrate promoters but not introns

Our data revealed that nCpG values follow a bimodal distribution at the promoter regions of genes in painted turtles (Fig. 1), consistent with most major vertebrates lineages studied ([56], Table 3), but not with platypus [29] or tunicates [27]. However, some interesting differences exist among bimodal patterns between turtles and other vertebrates. For instance, the CpG bimodality is less pronounced in turtle promoters than in human and chicken (Fig. 1 and [27]). Namely, the low- and high-CpG modes of the turtle promoter distribution overlap more extensively and are centered around lower CpG values than those of human and chicken promoters (Fig. 1 and [27]), suggesting a potentially larger role for DNA methylation as transcriptional regulator in reptiles than previously anticipated (i.e., greater historic methylation in turtle than in human and chicken). In human, high-CpG promoters are more abundant than low-CpG promoters, whereas the opposite is true in turtles (Fig. 1 and [27]). Human high-CpG promoters are hypermethylated less frequently whereas low-CpG promoters tend to be hypermethylated more often [28, 29], whereas differentially methylated genes in turtles had lower CpG content (Fig. 2b). The full implications of the observed methylation patterns in turtles are unknown. For instance, human promoters are methylated in somatic as well as germline cells and thus could be heritable [55]. Further, the CpG observed/expected ratio [CpG(O/E)] affects gene expression levels and breath in humans [57]. But further research is needed to test if the same is true in turtles. Unlike promoters, the bimodal nCpG content of turtle introns (Fig. 1) differs from other vertebrates, as it is unimodal in fish, amphibian, lizard, bird and human [27], and more strikingly bimodal in tunicates [27]. Subtle bimodality can pass undetected during qualitative evaluations (e.g., platypus [29]), unless it is explicitly tested statistically (this study and [27]). Notably, recent evidence suggests bimodal gene body methylation exists in mammals, birds and fish [58].

**Table 3:** Summary of exemplar studies exploring the diversity of nCpG distributions in vertebrate genomes. *Sources*: 1 = Weber et al. [28]; 2 = Elango et al. [27]; 3 = Yang et al. [29]

Group	Species	Region profiled	nCpG distribution	Source
*Mammals*	*Homo sapiens*	Promoters	Bimodal	1, 2, 3
		Introns	Unimodal	2
	*Pan troglodytes*	Promoters	Bimodal	3
	*Gorilla gorilla*	Promoters	Bimodal	3
	*Pongo abelii*	Promoters	Bimodal	3
	*Macaca mulatta*	Promoters	Bimodal	3
	*Monodelphis domestica*	Promoters	Bimodal	3
	*Mus musculus*	Promoters	Bimodal	3
	*Ornithorhynchus anatinus*	Promoters	Unimodal	3
*Birds*	*Gallus gallus*	Promoters	Bimodal	2, 3
		Introns	Unimodal	2
*Reptiles*	*Chrysemys picta*	Promoters	Bimodal	This study
		Introns	Bimodal	This study
		Exons	Bimodal	This study
		Intergenic	Bimodal	This study
*Amphibians*	*Xenopus tropicalis*	Promoters	Bimodal	2
		Introns	Unimodal	2
*Fish*	*Danio rerio*	Promoters	Bimodal	2
		Introns	Unimodal	2
*Tunicates*	*Ciona intestinalis*	Promoters	Unimodal	2
		Introns	Bimodal	2

The overall turtle CpG depletion agrees generally with vertebrates (where it is correlated with high DNA methylation) compared to invertebrates [19, 59, 60]. The overall distribution of nCpG values in the *C. picta* genome, disregarding bimodality, is higher for exons than for other regions (introns, promoters and intergenic sequences), suggesting lower exon methylation. In contrast, human exons are more highly methylated than introns [61]. CpG depletion in exons could affect transcription as in humans where it downregulates genes [62, 63]. Thus, we hypothesize that the higher nCpG content of *C. picta*’s exons relative to other regions (i.e., lower exon CpG depletion relative to overall depletion) might be the result of natural selection to preserve gene expression given that nCpG content is much lower in turtles than in other vertebrates, and perhaps also to prevent the accumulation of CpG to TpG mutations [19] that could produce non-functional proteins [64].

## nCpG content is a reasonable predictor of DNA methylation, except for tRNAs, and matches other vertebrates

Our results link DNA methylation at the resolution measured by MeDIP-seq and CpG depletion, consistent with humans [28] and insects [26]. Indeed, 94% of all the methylated genes revealed by MeDIP-seq had an nCpG ≤ 0.5, and 98.7% of all genes with nCpG ≤ 0.5 were actually methylated. Thus, CpG content is a reasonable indicator of methylation status, except for tRNAs. Namely, 90 of 182 annotated tRNAs were methylated, 40% of which (36/90) have an nCpG ≥ 0.8, indicating that CpG depletion has been suppressed historically in many tRNAs, perhaps to prevent CpG to TpG mutations [19]. This agrees with the high conservation of tRNA sequences in all domains of life [65]. Comparable methylation levels were found here (57% of the genome, including 78% of all CpG dinucleotides) as in fish, salamander, snake, birds and mammals using various approaches [24, 27, 31, 59, 66].

## Turtle methylation patterns suggest *cis*-regulation via DNA methylation

Sequences up to 50 kb upstream of genes in painted turtle, which should have potential regulatory roles, were methylated at significantly higher levels (46%) than gene bodies (40%) in hatchling gonads. Of these upstream windows, 16% fall within the boundaries of another gene upstream of the focal gene (within an intron in 94% of those cases), suggesting that perhaps some methylated genes may serve as alternative upstream promoters for downstream genes, as in humans [67, 68]. Importantly, DNA methylation in promoters is linked to gene silencing, whereas methylation in gene bodies may modulate transcription [8, 69], but further research is needed to test these hypotheses directly in turtles.

## DNA methylation varies by sex, gene and gene region

Results from our biological duplicates, which combined five males or five females each, were fairly consistent (Fig. 2), strengthening the inferences of sex-specific methylation. However, differences were also detected within sex, likely revealing genetic variation among individuals associated with DNA methylation (Fig. 3), but without obscuring some evident sex-specific methylation patterns.

Interestingly, 3% (541) of all methylated genes contained multiple differentially methylated windows, some hypermethylated in females while others in the same gene were hypermethylated in males (Fig. 2d). Introns showed the highest log-fold change in methylation between the sexes, followed by promoters and finally exons (Fig. 2c), but it is unclear whether turtle intronic methylation modulates transcription rather than silencing genes [8, 69], or whether it is linked to the transcription of antisense RNA, as observed in genes important for urogenital development in other vertebrates [70].

Because the males and females studied here were obtained at contrasting temperatures that produce a single sex (only males at 26 °C and only females at 31 °C), perhaps the sex-specific methylation in hatchlings is also temperature specific. Thermosensitive methylation of distinct windows within the same gene could potentially lead to alternative splicing of sex-specific transcripts, as in humans [71]. Alternatively, the observed differential methylation could be a consequence of sexual differentiation. Further studies are needed to test these alternative hypotheses.

## Known regulators of sexual development are differentially methylated in TSD turtles

We investigated 53 genes in the mammalian urogenital regulatory network, whose reptilian homologs are differentially expressed in TSD turtles, including *Wt1* [72, 73], *Sf1* [74, 75], *Dax1* [41, 76], *Sox9* [76–79], *Aromatase* [17, 80], *Dmrt1* [76, 78, 81], *Esr1* [82, 83] and *Rspo1* [79]. Of these, methylation has been studied only in *aromatase* and *Sox9* and demonstrated to influence transcription in developing TSD reptiles (slider turtle [17], American alligator [84]) and a TSD fish [15]. Our results provide evidence (1) that many more genes in this regulatory network are differentially methylated between male and female gonads, (2) that sexually dimorphic methylation persists post-hatching and (3) that it affects some genes that are differentially expressed during gonadal development.

Genes such as *Amh*, *Ar*, *Gata4*, *Lhx9* and *Sf1* that govern testicular formation are upregulated at male-producing temperature (MPT) late in the thermosensitive period of *C. picta* [42, 43] and displayed hypermethylated promoters in female hatchlings (Table 4). The exception was *Sf1*, whose introns were differentially methylated (Table 4). Additionally, three hypermethylated intronic windows in male hatchlings were observed in *Wt1,* a gene important for the formation of the bipotential gonad and later testicular development [85] that is upregulated at MPT early in the thermosensitive period but not by stage 22, and is a candidate TSD master gene [43, 72, 85]. The consequences of turtle intronic methylation remain unknown for alternative splicing, the use of alternative promoters, transcription of antisense RNA or transcription modulation [8, 64, 69, 70]. Data for *Amh*, *Ar*, *Gata4* and *Lhx9* suggest that upregulation in one sex may result from silencing by promoter methylation in the opposite sex, which was also true for *Wnt4* and *Emx2* that govern ovarian formation [47, 48] and were hypermethylated in male hatchlings. These types of regulation do not appear to be ubiquitous as some other genes in the gonadal development network were differentially expressed but showed no differential methylation. Interestingly, *Lhx1* and *Gata4* displayed female hypermethylation in promoters (1 window each) and introns (2 and 3 windows, respectively) suggesting a potentially more complex regulation by methylation of these elements (Table 4).

**Table 4:** Summary of differentially methylated (FDR cutoff: 0.05) genes in hatchlings that are putatively involved in reptilian gonadogenesis

Gene	Sex of hatchlings showing hypermethylation	Hypermethylated region	Sex of stage 22 embryos showing upregulation
*Amh*	Female	P, I	Male
*Ar*	Female	P, D	Male
*Gata4*	Female	P, I (3)	Male
*Lhx9*	Female	P	Male
*Sf1*	Female	I	Male
*Lhx1*	Female	P, I (2)	–
*Emx2*	Male	E	–
*Insr*	Male	I	–
*Wnt4*	Male	I	–
*Apc*	Male	I	–
*Igf1r*	Male	P	–
*Tcf21*	Male	D	–
*Six1*	Female	D	–

*Amh*, *Ar*, *Gata4*, *Lhx9*, *Sf1* are genes upregulated at the male-producing temperature (26 °C) during stage 22 of embryonic development [42] and hypermethylated at 31 °C. All other cells denote differentially methylated genes that are not upregulated in the opposite sex

*P* promoter, *I* intron, *E* exon, *D* downstream of last exon; () indicates the number of methylated windows if >1

Our findings suggest that methylation marks may be stable and persist post-hatching, perhaps longer-term as in mammals [86, 87]. Yet notably, *aromatase* showed no differential methylation in hatchlings, counter to observations in slider turtle embryos [17]. Assuming that embryonic methylation in slider and painted turtles is similar as is *aromatase* transcription [43, 75], our finding in *C. picta* hatchlings suggests that embryonic *aromatase* methylation may be transient, or alternatively, that it passed undetected by unknown technical limitations. Further research will help elucidate between these hypotheses directly.

A contrast of our turtle methylomes with those from the ZZ/ZW + TSD tongue sole fish [18] revealed the overlap of 22 genes (Additional file 1: Table S17) out of 56 genes differentially transcribed or methylated in tongue sole gonads (Additional file 1: Table S8 in [18]). Fish and painted turtle differed in overall patterns of methylation and expression, non-surprisingly given that fish transcription and that in other vertebrates has diverged profoundly [43]. The only exception was *Lhx9* which was upregulated at MPT in turtle embryos and was hypermethyled in both the tongue sole and painted turtle. Lastly, two other differentially methylated genes in turtle are worth mentioning, the epidermal growth factor receptor (*Egfr*) which has been previously implicated in sexual dimorphism in *Drosophila* [88], and *Mafb*, a gene with sexually dimorphic expression responsible for masculinization of male genitalia in mice [89].

## Potential thermal transmitters are differentially methylated in TSD turtles

Sexually dimorphic methylation was present in hatchling gonads in several gene candidates that may help convert the environmental temperature into sexual development signals to establish the sexual fate in TSD taxa [13, 36, 43, 90] (Additional file 1: Table S4, Additional file 1: Table S5, Additional file 1: Table S6, Additional file 1: Table S7, Additional file 1: Table S8, Additional file 1: Table S9, Additional file 1: Table S10, Additional file 1: Table S11), rendering them targets for future study. These include several heat shock genes, transient receptor potential genes, a number of kinases, androgen-/estrogen-related genes and histone-related genes. Namely, several *heat shock protein* genes (e.g., *Hspa4*, *Hspa12a* and *Hspa12b*) were hypermethylated in males, and they are differentially expressed by temperature in alligators and could play a role in TSD [36]. Further, the *cold*-*inducible RNA*-*binding protein* (*Cirbp*), a putative key TSD gene that is upregulated at FPT in snapping turtles *Chelydra serpentina* (TSD) [91] and *C. picta* [42], showed hypermethylated exons in females (Additional file 1: Table S4), perhaps causing differential splicing as in humans [71]. Additionally, *transient receptor potential genes* (e.g., *Trpm1*, *Trpm2*, *Trpm3*, *Trpm7* and *Trpm8*) can respond to temperature stimuli [92] and were differentially methylated. Moreover, many *kinases* such as members of the *Mapk* signaling family which helps activate *Sry* in mice [93, 94] were differentially methylated, along with many *androgen* and *estrogen* signaling genes. Also, genes involved in *histone modification* directly regulate transcription and can act in a sex-specific manner. For instance, *aromatase* transcription in embryonic gonads of *T. scripta* turtle embryos increases by demethylation [17] which depends on local histone acetylation in mammals [95]. We found hypermethylation of *histone acetyltransferases* (*Kat2a* and *Kat6a*) in males and of *deacetylases* (*Hdac4*, *Hdac7* and *Hdac8*) in females, but it is unclear whether differential methylation of histone modifiers is linked to sexually dimorphic transcription in turtles.

### Differentially methylated genes have undergone greater historic methylation

Intriguingly, differential methylation in *C. picta* hatchlings coincided with regions that showed significantly lower normalized CpG content relative to the genome-wide methylation levels, a pattern observed in both gene bodies and promoters (Fig. 2e, f) that indicates stronger historic methylation in these genomic regions. This pattern includes various genes in the sex determination/differentiation network and supports the notion that differential methylation of elements in this network has occurred over long periods during turtle evolution, leaving a footprint of historic methylation at the DNA sequence level [19, 20].

## Differential repeat methylation is linked to methylation of adjacent genes

Importantly, over 95% of methylated genes (but not unmethylated genes) were nearby methylated repeats. Further, repeat methylation was associated with sexually dimorphic methylation of neighboring genes, as >70% of repeat windows nearby differentially methylated genes were methylated in the same direction as the genes (Table 2). This suggests that repeat methylation could promote DNA methylation in adjacent genes perhaps mediating their transcription in *C. picta* as occurs in *Drosophila* and human [96, 97]. Repeat methylation may vary by repeat category and developmental stage as observed in fish [98], but further research is needed to test for DNA methylation in developing turtles.

However, we found that DNA methylation targets repeat elements in general more often than expected by their overall genome abundance, perhaps as a result of repeat silencing. Namely, 10% of the *C. picta* genome assembly is composed of transposable elements [37] [and 25% of the genome consists overall repeats (Additional file 1: Table S16)], whereas repeats composed 40% of all methylated regions. This result is consistent with findings in rat using our same approach where the high proportion of repeats in the methylome was attributed to repeat silencing (41% repeats in the rat genome and 53% in the rat methylome) [99]. Notably, insights about the relative abundance of repeat categories vary depending on whether the entire turtle genome was considered or only the genomic fraction that was composed of repeats (Fig. 4a, b). On the one hand, relative abundance among repeat categories in the methylome followed their relative abundance in the entire genome (Fig. 4a). For instance, CR1 repeats were the most abundant in the methylome, followed by HATs, DIRS, Gypsy and Harbinger repeats, just as in the painted turtle genome [37]), and in the transcriptome [42] (Fig. 4a). On the other hand, some repeat categories were overrepresented and others underrepresented in the methylome compared to their abundance in just the fraction of the genome composed of repeats (Fig. 4b). Thus, not fully conclusive evidence was found for overall repeat silencing by DNA methylation [64] in turtles as occurs in humans [100]. Interestingly, CR1 repeats concentrate at centromeres of a few *C. picta* chromosomes [38] such that their high methylome abundance may reflect chromosome stabilization by DNA methylation of centromeric repeats [64].

## Conclusions

Ours is the first genome-wide assessment of DNA methylation in reptiles and the first study of sexually dimorphic methylation levels in a purely TSD vertebrate [51]. As such, this study sheds light on the epigenetic modifications that may play a role in the development of phenotypically plastic vertebrates, complementing recent work on a fish with mixed sex determination [18]. Our MeDIP-seq data provide empirical validation of in silico predictions obtained from nCpG content never done before in reptiles and show that nCpG content is a reasonable predictor of actual methylation status at the resolution of MeDIP-seq. We found that painted turtles possess a unique pattern with nCpG values below those of other vertebrates, indicative of global and extensive historic methylation. In contrast, actual methylation levels given the turtle genome CpG content agree with those described for most vertebrates. Our data helped us identify several genes whose methylation status renders them candidates for a putative function in regulating transcription in a thermosensitive manner, a pattern that appears associated with methylation status of neighboring repeat elements. Based on this correlational pattern, we speculate that methylation of some of these elements may play a key role in mediating the sexual fate of TSD reptiles. Further research is warranted to test this hypothesis and the prevalence of DNA methylation in governing the sexual outcome in TSD turtles as it does in sex reversal of fish with mixed sex determination [18], or whether instead, most differential methylation observed in turtle hatchlings is the consequence of sexual differentiation and not its cause.

## Methods

### Sample collection

Eggs from several freshly laid clutches were obtained from a turtle farm, and equal numbers from each clutch were assigned randomly to incubators set at 26 and 31 °C that produce 100% males and 100% females, respectively, under standard incubation conditions [101]. Turtle embryos are at gastrula stage at oviposition [102]. All hatchlings were raised for 3 months in tanks with water heated to ~26 °C and fed ad libitum, to allow full gonadal differentiation prior to sexing. Individual sex was diagnosed by gonadal inspection [51, 103]. All procedures were approved by the IACUC of Iowa State University.

### DNA isolation and sequencing

We extracted DNA from the gonads of 3-month-old *C. picta* hatchlings (ten males and ten females, and pooled in groups of five hatchlings for replication) using the Gentra Puregene DNA extraction kit (Gentra) following the manufacturer’s instructions. DNA was processed by BGI Americas following the MeDIP protocol [104] where DNA was fragmented and denatured, and the methylated DNA was immunoprecipitated using the MagMEDI kit (Diagenode). A positive control (methylated DNA) and a negative control (unmethylated DNA) were used at the MeDIP step to ensure that the MeDIP worked properly. The methylated DNA was size selected (100–300 bp) and sequenced using the Illumina HiSeq paired-end protocol. We obtained between 126 million and 163 million 50-bp reads per library amounting to a total of ~564 million reads (Table 1).

### Methylome construction and differential methylation analysis

We mapped the sequencing reads to the *C. picta* genome version 3.0.1 [37] using Bowtie2 version 2.2.5 [105]. We filtered out the unmapped reads using Samtools [106]. We used the MEDIPS package [107] in four steps: (a) to build an index for the *C. picta* genome and to ensure fast querying of the alignment files. (b) To model read counts under a negative binomial distribution. (c) To quantify mapped read counts per 500-bp windows in RPKM (reads per kilobase of genes per million mapped reads). Mean methylation levels were calculated for each 500-bp window by averaging the corresponding RPKM levels in the replicates. A gene was considered as methylated if it contained one or more methylated windows within its start and end coordinates. (d) To merge methylated windows and compute differential methylation by sex, while controlling for false discoveries [108] at a *q*-value cutoff =0.05. Any gene with a *q*-value <0.05 was considered as differentially methylated. Besides the positive and negative controls used during the MeDIP procedure described above, primers were also designed to validate by PCR the differential methylation identified by MeDIP-seq for the gene *Fezf2* that was hypermethylated in females compared to males [109]. The primer cocktail consisted of one forward primer (F1: 5′-GGG GTG AAA AAC CAC AG-3′) and two reverse primers, one closer to the F1 (R1: 5′-CAC ACA CAA GGA GG-3′) and one further away (R2: 5′-CAG CAA CAA CTT GAT TTG G-3′) (Fig. 3). Primers F1 + R1 flank a shorter non-methylated area that should amplify in any individual, which is nested within the larger region encompassed by F1 + R2 that contains the differentially methylated area, and which amplifies only when methylation protects the DNA from digestion by a methylation-sensitive restriction enzyme (HpaII in our case). Gonadal DNA from three male and three female hatchlings was digested with HpaII for 30 min at 37 °C. PCR was carried out in 15ul reactions, using 1ul of 100 ng/ul undigested DNA (control) or digested DNA (experimental) as template, and 5ul PCR products along with 1ul 1 kb plus DNA ladder (Invitrogen) were visualized in 1% agarose gels stained with ethidium bromide. The PCR cocktail contained 1.5 mM MgCl2, 0.2 mM dNTPs, 0.4uM of each primer (F1, R1, R2), 0.4U Taq polymerase, ~100 ng DNA, 1.5ul 10X buffer and 10.5 μl water. PCR conditions included initial denaturing at 94 °C for 3 min, followed by 35 cycles of denaturing at 94 °C for 30 s, annealing at 58 °C for 30 s, and extension at 72 °C for 1 min.

We used Kolmogorov–Smirnov tests to determine whether the nCpG content distribution of all annotated genes was comparable to those identified by the MEDIPS package. To test whether the nCpG of the differentially methylated genes varied significantly from that of all the methylated genes, we performed a resampling test [110] by iteratively drawing a random subset of genes (equal to the number of differentially methylated genes) from the entire set of methylated genes. We used RepeatMasker v3.3.0 [111] to identify repeats in the *C. picta* genome. We used pairwise Kruskal–Wallis tests [112] to test whether repeat abundances from the genome, methylome and transcriptome came from the same population. We used Bedtools v2.17.0 [113] to compute repeats overlapping with methylated regions identified by MEDIPS. We used permutation tests to evaluate differences in the abundance of methylated DNA repeats occurring in vicinity of methylated genes versus non-methylated genes. In the absence of transcriptional data from hatchlings, we used our transcriptomic dataset from late-developing embryos (stage 22) from another study [42] to test for an association between methylation patterns in hatchlings and transcription patterns using Fisher’s exact test. For this purpose, a contingency table was generated of genes that were differentially expressed (or not) and differentially methylated (or not), and a hypergeometric distribution was used to then determine the p value. A p value of ~1 would indicate no significant association between differential methylation and differential expression. We used R [114] to perform regression tests to model transcriptome abundance using genomic abundance of repeats and methylated repeats. We used the DAVID Bioinformatics knowledgebase [115] to assess the enrichment of functional categories to which the differentially methylated genes belong.

### Analysis of normalized CpG content

The normalized CpG content (nCpG) is calculated as:

$${\text{nCpG}} = \frac{{\left( {\frac{cg}{l}} \right)}}{{\left( {\frac{c}{l}} \right) \times \left( {\frac{g}{l}} \right)}}$$

for a sequence of length *l*, where *c* is the number of occurrences of cytosine, *g* is the number of occurrences of guanine and *cg* is the number of times cytosine is bordered by guanine linked by a phosphate group (CpG) [21]. In theory, nCpG values can range from 0 to +infinity for an infinitely long sequence, with a value of 1 when the number of CpG dinucleotides observed is equal to the expected based on the sequence length and abundance of C and G. Values <1 denote CpG depletion from what is expected by chance, and values >1 represent overabundance of CpGs from random expectation. The methylation of CpG dinucleotides initiates the deamination of the cytosine, transforming it to thymine, thus lowering the nCpG to <1. Studies conducted in vertebrate and invertebrate animals reveal an upper limit between 2 and 2.5 for various genomic regions [21, 25–27, 29, 116].

We used Bedtools [113] to parse the exon, intron, promoter and intergenic coordinates from the *C. picta* genome [37], given the annotations in gff3 format. We used in-house perl scripts to compute the CpG contents by genomic region. For the nCpG distributions, the R package Mclust [39] was used to assess likelihood of a mixture model with one (G = 1) and two (G = 2) components, with the better fit model decided by a likelihood ratio test.

## Additional file

**Additional file 1. Table S1:** Genes with distinct hypermethylated sex-specific windows within the same gene. **Table S2:** Categories enriched (*p* = 0.05) in hypermethylated genes at male-producing temperature (26 °C). **Table S3:** Categories enriched (*p* = 0.05) in hypermethylated genes at female-producing temperature (31 °C). Tables S4 through S11—differentially methylated genes in *C. picta* hatchling methylomes along with RPKM, fold change and edgeR *p* value: **Table S4:** Heat shock genes. **Table S5:** Androgen-/estrogen-related genes. **Table S6:** Kinases. **Table S7:** Histone-related genes. **Table S8:** Ubiquitin-related genes. **Table S9:** Transient receptor potential genes. **Table S10:** Genes involved in cell proliferation. **Table S11:** Germline-related genes. **Table S12:** Number of differentially methylated genes by GO term present in testis and ovaries of *C. picta* hatchlings. **Table S13:** Comprehensive list of differentially methylated genes and associated GO pathways between testis and ovaries of *C. picta* hatchlings. **Table S14:** Statistics of all differentially methylated genes between testis and ovaries of *C. picta* hatchlings. **Table S15:** Genes differentially expressed in the *C. picta* transcriptome (stage 22) [42] and differentially methylated in the hatchling methylome (this study). **Table S16:** Relative abundance distribution of repeat categories as a fraction of the genome. **Table S17:** Overlapping genes between *C. picta* hatchling methylomes and the methylomes of tongue sole [18].

## Abbreviations

*Amh*: anti-mullerian hormone
*Ar*: androgen receptor
*Cirbp*: cold-inducible RNA-binding protein
CR1: chicken repeat 1
*Dax1*: dosage-sensitive sex reversal, adrenal hypoplasia critical region, on chromosome X, gene 1
*Dmrt1*: doublesex and mab-3-related transcription factor 1
*Egfr*: epidermal growth factor receptor
*Emx2*: empty spiracles homeobox 2
*Esr1*: estrogen receptor 1
*Gata4*: gata binding protein 4
GSD: genotypic sex determination
HAT: transposon superfamily named after Hobo from *Drosophila melanogaster*, Ac from maize and Tam3 from the snapdragon
*Hdac**x*: histone deacetylase number x
HiSeq: high-throughput sequencing technology by Illumina
HPLC: high-performance liquid chromatography
*Hsp**x*: heat-shock protein number x
*Lhx1*: lim homoeobox 1
*Lhx9*: lim homoeobox 9
*Mafb*: MAF BZIP transcription factor B
*Mapk*: mitogen-activated protein kinase
MeDIP-seq: methyl DNA immunoprecipitation sequencing
nCpG: normalized CpG content
RPKM: reads per kilobase of genes per million mapped reads
*Rspo1*: R-spondin1
*Sf1*: steroidogenic factor 1
*Sox9*: SRY (sex-determining region Y)-box 9
*Sry*: sex-determining region Y
tRNA: transfer RNA
*Trpm**x*: transient receptor potential cation channel subfamily M member number x
TSD: temperature-dependent sex determination
*Wnt4*: wingless-type MMTV integration site family, member 4
*Wt1*: Wilms tumor protein 1
